# Written Prescriptions

**Published:** 1867-09

**Authors:** 


### Editorial Department.

### Written Prescriptions.

In all the larger cities, physicians have adopted the plan of making a written prescription, and of entrusting the compounding as well as the selection of the material of which it shall be composed, to the druggist. It is certainly a very great relief to the busy practitioner to thus escape the labor of compounding his remedies; and where both physician and druggist perform their parts faithfully and intelligently the harmony of the transaction is sufficiently perfect. It can hardly be possible that this custom, now so universal, can be essentially changed, and perhaps it is not desirable for any of the parties concerned, that any important change be made, even, if possible; but it will be readily apparent how nearly vital to the reputation of the physician is the faithfulness and fidelity of the pharmaceutist; remedies, however judiciously prescribed, if poorly compounded, and of inferior quality, are useless. The odium of failure does not rest with the medicine and druggist, but falls back upon the physician; it is to him the patient looks for relief, and when the prescription is filled, it is medicine from Dr. ——— and not from anybody's drug store. There is great pains taken in the elegance and neatness with which prescriptions are dispensed; this is essential to business success, and is really commendable; but a nice label and a neatly done up package cannot compensate for inferior medicine, or unskillful composition, though it must be confessed that taste and elegance in the exterior, is presumptive evidence of purity and genuineness within.

The pharmaceutist who is skilled in his art, is entitled to adequate compensation for his time and labor, and there can be no fair-minded individual but will readily concede this point. There is, however, another view to be taken which must not be wholly lost sight of in canvassing this question.

The expense of medicine to those who are poor, when also sick, is certainly to be considered, and, indeed, those who are not what may be called poor, often feel when sick or disabled for any great length of time, that they cannot afford the cost of medicine. At the present time we have an immensely expensive way of making up our remedies, which was not common formerly, when physicians supplied their patients, generally without extra charge. We have sometimes thought how much better on their account it would be for patients, if this practice still prevailed, and how unfortunate for the drug trade. The manufacturing chemists have done much to improve the elegance and agreeableness, as well as increase the cost of medicine, but have added nothing to its simplicity, and probably in many instances nothing to its efficiency. They have multiplied the number of articles of medicine indefinitely, and mixed and sugar-covered all the important and valuable substances we use in the treatment of disease, until we hardly know anything of the substances we prescribe, except what is stated by the manufacturer upon the label, which may, or may not, give the names of the medicines and proportions which each parcel contains. If we give iron, our patients pay its weight in gold; if bark or bismuth or strychnine, or indeed any other medicine is prescribed in the present elegant combinations in which it is offered, the cost is so vastly increased beyond human conception, that most people must regard it, considering its price, as the famed nectar, fit drink for gods. The elixir calasaya, iron and bismuth, is an elegant and useful medicine, and named as a sample of our present materia medica—is an elegant and agreeable beverage, useful as a tonic, and much less likely to disturb digestion than most mixtures used for strengthening purposes. It is well enough for the wealthy to indulge in such luxuries, but most sick people require relief by simpler and cheaper means. The tonic remedies, by which is usually meant the bitters and iron, combined with spirits, are the harmless medicines, which those who have been and some who still are sick, take eternally, until their own and their friends resources are exhausted. It is not very uncommon to find people who have scarcely the common articles of food for themselves and children, supplied with calasaya cordial or conium and iron mixture, obtained at fabulons prices, upon the idea that strength is to be gained out of these articles. We have even known mothers set aside milk, and buy "chemical food" for nursing children, as if the Almighty had made a mistake in providing nourishment for infancy; and worse still, we have known this plan adopted upon recommendation of attending physician. Meat and vegetables and milk, the only real strengthening medicines yet known, are never mentioned as tonics. This probably grows out of the fact that their active principles are not profitably extracted, and they are not advantageously mixed up with sugar and spirits in agreeable proportions. The public may not know what is the best tonic; physicians should know that food is almost the only tonic, is certainly the best and most indispensable support. This is suggested only, of a condition of things which requires attention.

Many physicians are perhaps ignorant of the influence our present system of prescribing has upon a great many individuals and families. Their philosophy is this: If we call the physician, in our opinion most worthy our confidence, his prescription is to be filled at the druggists. He always sets the highest estimate upon his own services, and his prescription often costs more than his advice. If we are reasonably safe in calling, some medicine monger of the regular stripe, or some monger of no medicine mould, who will carry what he gives, or rather give what he carries, it will be an economy, at least, worthy of trial; we can go back if we find necessary to the old and true. With something of this reasoning a great many unsettled, unthinking people, who would not be without a show of care when sick, even if the substance is wanting, go over to the realms of the shadowy and intangible; and some of the otherwise sensible public go with them. It is a matter of some importance to physicians that patients receive such remedies as necessary when actually sick without too great expense, and this matter is of importance on many accounts. We shall not intimate that medicines are sold at too great profit, but we propose to say that the necessary and valuable remedies when prescribed in simple form, are both more reliable and less expensive than when combined, compounded and confounded, with each other and with everything else, to suit the ingenuity of the trade. It is largely with physicians to correct a condition which has something to do with the consumption of sugar of milk, and which has other and more important objections than any which could be urged from this source. How often do our patients, even those in well off circumstances, object to the great cost of medicines, and how often do we find the poor unable to meet the demand. The remedy is with us, more than with druggists, and a moment of reflection will show every physician how he can adapt his prescriptions to the wants and necessities of his patients. If a physician puts nearly all the articles of the materia medica into one mess, by the rule of some obsolete prescription, he must expect not only wonderful results, but corresponding prices; and his victims must suffer the consequences. We believe that such prescriptions, quite too common, should be paid for, and it might be well if government should require also upon the bottle an *Internal Revenue stamp* (?) But a common remedy, containing twelve grains of Dover's powder, or the same amount of opium, or a few compound colycinth pills, should not be a very expensive parcel under ordinary circumstances. The custom of charging the same for fluid medicines by the ounce is manifestly unfair, for every one will see that they vary in value to the widest extent. Water is very cheap in Buffalo, and we presume in other towns and cities situated upon navigable lakes and rivers, the same remark may be generally true. If it is put up in bottles, to any great extent, at fifteen cents per ounce, and without a very expensive label, there mnst be a handsome profit realized upon it. We would simply call the attention of druggists to the obvious truth in this matter, though doubtless all have considered it sufficiently without any invitation from us. We wish, however, in this connection to say for the druggists of Buffalo, that in our opinion more intelligent, high-minded, faithful and trusty men cannot be found in the pursuit of any business or profession, taking them as a body; and that the medical profession in our city are under obligations to them for the faithfulness and fidelity with which they dispense their remedies, and the care and intelligence generally used not only in compounding and dispensing, but also in selecting articles of the best quality. Having said this much in their favor, they will forgive whatever may be said which is not so complimentary, though we have no occasion to propitiate their feelings. The relations between the physician and druggist are so intimate, that their interests may be said to be identical, and that harmony and good will are essential to both. We should regard any intimation derogatory to the business character of druggists in Buffalo, as a personal assault, and would defend them, as much as possible, with the same feelings that we would sustain and support a member of the medical profession.

Some physicians in the city, and all in the country, dispense more or less their own remedies; generally in the city this is not feasible, for many families, even who can ill afford to gratify their fancy, prefer going some distance and paying liberally, receiving their powders in uniform papers, and nicely labeled box, to taking them gratuitously from their physician, when done up in his usually hurried and inelegant manner. Again, the busy physician who wishes to make calls as rapidly as possible. and whose time is fully employed, cannot afford to make up his prescriptions; he must delegate this part of his duty to another; to no one so appropriately as to to the druggist. Physicians who can dispense their own remedies, and who from situation must do it, may congratulate themselves and patients upon their "lines having fallen to them in pleasant places."

The force of all this canvass of the custom of writing prescriptions is this:—We have lost sight of the real articles in medicine by its admixture, concentration, combination and general change. We consequently are less simple, less safe, and less sensible in our means of cure, and our patients take fancy medicines largely, more to test the pharmaceutical cookery of the present time, than for cure of any actual disease. Reform requires no organization or machinery of any kind. A common sense view of the condition and a common sense mode of action, will *make necessary medication* within reach of nearly all, while the other division of practice may safely be allowed to drift *fashionably* its own way.

